# Fast-Track surgery protocol in perioperative care for gynecological laparoscopy

**DOI:** 10.12669/pjms.40.7.9117

**Published:** 2024-08

**Authors:** Hongping Zhu, Xiaoying Xu

**Affiliations:** 1 Hongping Zhu Department of Obstetrics and Gynecology, Suzhou Hospital of Integrated Traditional Chinese and Western Medicine, No. 39 Xiashatang, Mudu Town, Wuzhong District, Suzhou City, Jiangsu Province 215101, China; 2 Xiaoying Xu Department of Obstetrics and Gynecology, Suzhou Hospital of Integrated Traditional Chinese and Western Medicine, No. 39 Xiashatang, Mudu Town, Wuzhong District, Suzhou City, Jiangsu Province 215101, China

**Keywords:** Fast-track surgery, Laparoscopic gynaecological surgeries, Perioperative care, Nursing

## Abstract

**Objectives:**

This study aimed to compare fast-track surgery (FTS) and traditional perioperative care protocols in laparoscopic gynecological surgeries, assessing their impact on length of stay (LOS), recovery time, and postoperative complications.

**Methods:**

A case-control retrospective study was conducted at Suzhou Hospital of Integrated Chinese and Western Medicine, involving 167 patients undergoing laparoscopic gynecological surgery from June 2021 to June 2023. Of them, 81 patients underwent surgery based on the FTS protocol (FTS group) and 86 patients received a traditional perioperative management (control group). Patients in both groups underwent gynecologic laparoscopic procedures, including uterine, ovarian and tubal surgeries. Data were collected on general patients’ characteristics, including age, BMI, surgery type and time, intestinal recovery and out-of-bed activity time, LOS, pain levels, and postoperative complications. Wilcoxon rank sum test with continuity correction was used to assess the difference in operative characteristics and postoperative pain levels. Fisher’s exact test was used to assess the difference in overall frequency of postoperative complications between groups.

**Results:**

Patients in the FTS group exhibited faster intestinal recovery, shorter mobilization time, and reduced LOS compared to the control group. Pain levels were significantly lower at one, six and twelve hours post-surgery in the FTS group. Overall, the proportion of postoperative complications was significantly lower in the FTS group than in the control group.

**Conclusions:**

Implementing the FTS protocol in laparoscopic gynecological surgeries for benign conditions can reduce LOS, accelerate recovery, and minimize pain without increasing postoperative complications. Further research with more diverse patient populations is warranted to validate these findings.

## INTRODUCTION

Fast-track surgery (FTS) is a comprehensive surgical and nursing approach aimed at accelerating patient recovery, minimizing stress reactions, decreasing postoperative complications and increasing patients’ satisfaction. The method was first introduced in the late 1990s by Bardram et al. and Kehlet et al. for colonic surgeries, and since then, has been extensively applied in many surgical procedures, including major cardio-thoracic operations.^1^–^3^ FTS approach aims to reduce the length of hospital stay (LOS) and related burden on the healthcare system. A recent meta-analysis demonstrated that FTS approach can reduce LOS by an average of 2.35 days and reduce healthcare expenses by $639.06 per patient.^4^

FTS protocol focuses on preventing and minimizing various factors that can negatively affect patient recovery following surgical procedures. These factors include pain and the excessive use of opioids for pain management, postoperative nausea and vomiting (PONV), paralytic ileus, fatigue, and sleep problems. The primary objective of the FTS protocol is to optimize patient outcomes by addressing and mitigating these specific factors throughout the perioperative period. The FTS protocol includes preoperative preparation composed of patient education aimed at reducing the anxiety level and decreasing the length of preoperative fasting compared to the traditional surgery methods.^5^,^6^ Intraoperative nursing following the FTS protocol focuses on optimal pain control using short-acting anesthetics during the surgery while minimizing opioid-related side effects^7^ promoting rapid emergence from anesthesia, which reduces overall recovery room time.^8^ Finally, postoperative FTS nursing focuses on early enteral feeding and early mobilization as a critical component of rapid rehabilitation after surgery.^9^Embase, and the Cochrane controlled trials register The Enhanced Recovery After Surgery (ERAS®) Society has recently published a detailed FTS strategy for gynecology and gynecological oncology. 10

The primary objectives of this research were to compare FTS and traditional perioperative care protocols in laparoscopic gynecological surgeries and examine the following theories: H0: There is no difference in the length of stay, recovery time and postoperative complications between patients who were treated based on FTS or the traditional management protocol. H1: The length of stay, recovery time, and frequency of postoperative complications differ between the two groups.

## METHODS

A total of 167 patients who underwent gynecological laparoscopy at Suzhou Hospital of Integrated Chinese and Western Medicine from June 2021 to June 2023 were included to the study.

### Ethical Approval

The ethical approval was obtained from the institutional ethics committee (No. 2023-005, Date: 2023-08).

The inclusion criteria were the inpatients aged 18-70 years who received gynecological laparoscopy for benign conditions, performed under general anesthesia. The presence of full clinical data was required. Patients with malignant gynecological tumours, conversion from laparoscopy to open surgery, cardiopulmonary dysfunction or other important organ diseases, blood diseases, diabetes, severe psychological or neurological disease and other serious complications were excluded. The patients with acute gynecological abdomen undergoing emergency surgical treatment and the ones having previous history of abdominal surgery were also amongst the exclusion criteria. Of included, 81 patients underwent surgery based on the FTS protocol (FTS group) and 86 patients received a traditional nursing intervention (control group).

### Traditional nursing intervention:

Patients in the control group received traditional nursing interventions that included close monitoring of the health of patients before the surgery, explaining to patients the knowledge and precautions related to laparoscopic surgical treatment, and ensuring cooperation of patients and their families with various procedures of the surgery. Patients were required to strictly fast for 6 h before the operation, drink 300 ml of carbohydrates (Compound Polyethylene Glycol Electrolyte Dispersion (II) [trade name: He Shuang, Shenzhen Wanhe Pharmaceutical Co.] 4 h before the operation and abstain from drinking thereafter. During the operation, the nursing staff strictly cooperated with the anaesthesiologist and surgeon’s requirements, closely observed the changes of the patient’s vital signs and recorded them accurately. After the operation, the nursing staff paid close attention to any changes in patient’s condition, and wound healing, and followed doctor’s instructions to ensure appropriate pain management. Patient were given detailed instructions about postoperative rehabilitation.

### FTS nursing interventions:

The observation group received pre-, intra- and post-operative fast track surgical nursing intervention on the basis of the traditional nursing intervention

### Preoperative:

One day before the patient’s operation, the informed consent about the FTS nursing interventions was provided.

### Intraoperative:

Temperature of the operating room was maintained at 24~46 °C, humidity maintained at 50.0%~60.0%, and patients received intrathecal anesthesia. During the operation, extra measures were taken to ensure patient’s heat preservation in order to effectively reduce the stress caused by low temperature, and included infusion of pre-warmed fluids to ensure that the patient’s body temperature is maintained at 36 °C or so, and a special heater if needed. Nursing staff ensured strict control and timely adjustment of infusion volume and rate to maintain normal blood pressure. All changes in the vital signs of the patient during the operation were monitored and recorded, and body surface temperature was regularly measured to better control the temperature of the operating theatre. Nursing personnel paid attention to the individual status of each patient, and took targeted nursing measures when needed.

### Postoperative:

According to the patient’s condition and under the anesthesiologist’s guidance, the nursing staff used intravenous self-control analgesic pump to control patient’s analgesia for 48 hours. Psychological interventions were given to the patient, and attempts were made to lower patient’s pain sensitivity by distraction (such as music) and medication. In terms of diet, the patients were instructed to drink an appropriate amount of water after six hour after the operation, and if no obvious discomfort was noted, a certain amount of unsweetened non-dairy fluid was allowed. Patients could resume normal diet only after their bowel sounds returned to normal. In terms of postoperative activities, the patients were instructed and assisted to perform ankle pump exercise every 15 minutes and turn over. Deep breathing exercises and coughing was done every two hour At six hour postoperatively, patients were guided to carry out independent activities in bed, and were assisted to walk on the ground for two hour after twenty fur hours. The urinary catheter was removed within 24 hour postoperatively.

Data collection was conducted by completing a specifically designed questionnaire. General characteristics of patients were obtained, such as age and body mass index (BMI); intra- and post surgery variables included type of surgery, time in surgery, volume of intraoperative bleeding, time of first out-of-bed activity, LOS and intestinal recovery time including time of first bowel movement after extubation, recovery time of intestinal sounds and first exhaust time. The pain was evaluated using a visual analog scale (VAS) that ranged from 0 (no pain) to 10 (most severe pain) at one, six, twelve, and twenty four hours post-operation. Postoperative complications were recorded during the first 30 days after the surgery and included nausea and vomiting (PONV), severe bleeding, pulmonary or urinary tract infection and poor wound healing.

### Statistical Analysis:

The continuous demographic and clinical characteristics were tested for normality using the Shapiro-Wilk test. Following the test results, which are not included here, we summarized all continuous characteristics using the median and interquartile range (IQR), as customary for variables that do not follow a normal distribution. The Wilcoxon rank-sum test was employed to examine the difference in the continuous variables between the study groups. The categorical variables were summarized as frequencies and proportion of cases in each study group. Pearson’s chi-squared test was used to assess the difference in surgery types between groups. Fisher’s exact test was utilized to determine the difference in frequency of postoperative complications between groups. All statistical analyses were performed using R language version 4.2.2, and a p-value < 0.05 was considered statistically significant.

## RESULTS

One hundred sixty-seven patients who underwent gynecological laparoscopic surgery for benign conditions were enrolled in the study. Of them, 81 patients underwent surgery based on the FTS protocol (FTS group) and 86 patients received a traditional perioperative management (control group). As summarized in Table-I, patients’ age, BMI, and surgery types were comparable between the two groups (p > 0.05).

**Table-I:** Baseline characteristics of participants in FTS protocol and control group.

Parameters	FTS protocol (n = 81)	Control (n = 86)	Test statistic^$#^	p-Value
***Baseline characteristics^$^, Median (IQR)***				
Age, years	52 (48-59)	52 (48-59)	3596	0.72
BMI, kg/m²	23.5 (20.5-25.5)	22.5 (20.5-24.5)	4053	0.07
***Surgery type^#^, n (%)***				
Uterine surgery	34 (42)	38 (44.2)	1.67	0.89
Ovarian surgery	20 (24.7)	18 (20.9)		
Extrauterine pregnancy surgery	10 (12.3)	14 (16.3)		
Tubal surgery	7 (8.6)	5 (5.8)		
Uterine suspension surgery	6 (7.4)	5 (5.8)		
Uterine scar pregnancy surgery	4 (4.9)	6 (7)		

$ Wilcoxon rank sum test with continuity correction was used to assess the difference in baseline characteristics.

# Pearson’s chi-squared test was used to assess the difference in surgery types between groups. BMI: body mass index.

The average operating time was 105 minutes, and the median estimated blood loss was 89 ml, with no significant differences observed between the FTS and the control group (Table-II). Patients in the FTS group had a significantly reduced time for intestinal recovery compared to the control group, as indicated by three criteria: first bowel movement time after extubation, intestinal sounds recovery time, and first exhaust time. All three signs of bowel recovery were observed significantly faster in the FTS group (22-40% decrease in time, p < 0.001). The time of postoperative mobilization in the FTS group was also significantly shorter compared to the control group: median time of first time out of bed activity was 17 and 25 hours, correspondingly (p < 0.001).

**Table-II:** Operative and postoperative characteristics of participants in FTS protocol and control groups.

Parameters		FTS protocol (n = 81)	Control (n = 86)	Test statistic^$#^	p-value
***Perioperative characteristics^$^, Median (IQR)***					
Time in surgery, min		105 (89-123)	105 (92-125)	3383.5	0.75
Intraoperative bleeding volume, ml		88 (81-101)	89 (71.25-103)	3554	0.82
Recovery time of intestinal sounds, hrs		10 (9-13)	14 (12-17)	1554.5	<0.001*
Exhaust time, hrs		15 (12-20)	25 (16.25-32)	1818	<0.001*
First bowel movement time after extubation, min		58 (52-65)	74 (65-83.75)	1107.5	<0.001*
First time out of bed activity time, hrs		17 (14-23)	25 (18-29)	1850	<0.001*
LOS, days		6 (5-6)	8 (7-9)	1120	<0.001*
***Level of pain based on VAS criteria^$^, Median (IQR)***					
VAS at 1 hour post-surgery		1 (1-2)	2 (1-2)	2101.5	<0.001*
VAS at 6 hours post-surgery		2 (1-2)	2 (2-3)	2104.5	<0.001*
VAS at 12 hours post-surgery		3 (2-3)	3 (3-4)	2186	<0.001*
VAS at 24 hours post-surgery		2 (1-2)	2 (1-2)	3294	0.50
***Postoperative complications^#^, n (%)***					
Overall complications		11 (13.6)	33 (38.4)	2.86	0.005*
Nausea and vomiting		5 (6.2)	13 (15.1)	2.69	0.08
Pulmonary infection		3 (3.7)	7 (8.1)	2.29	0.33
Urinary tract infection		1 (1.2)	6 (7)	5.95	0.12
Poor healing		1 (1.2)	4 (4.7)	3.87	0.37
Bleeding		1 (1.2)	3 (3.5)	2.88	0.62

* p-value statistically significant,

$ Wilcoxon rank sum test with continuity correction was used to assess the difference in operative characteristics and postoperative degree of pain,

# Fisher’s exact test was used to assess the difference in frequency of postoperative complications between groups, VAS: visual analog scale; LOS: length of stay.

FTS was associated with a notable 25% decrease in median LOS after surgery *versus* the control group (six vs. eight days, p < 0.001). Patients in the FTS group reported significantly lower pain levels at one, six and twelve hours after the surgery. However, at 24-hours post-surgery, both groups reported similar pain levels.

Overall, the proportion of postoperative complications was significantly lower in the FTS group than in the control group (13.6% and 38.4%, respectively, p = 0.005). Individual analysis of each type of postoperative complication demonstrated no statistically significant differences between the two study groups.

## DISCUSSION

Our study confirms that implementing an FTS approach in laparoscopic gynecological surgery for benign conditions is safe and does not increase the postoperative complications rate. FTS was associated with reduced time for intestinal recovery, shorter time to postoperative mobilization, decreased LOS and significantly lower pain levels at one, six and twelve hours after the surgery.

Our results are consistent with other reports showing that FTS method of perioperative care is associated with lower LOS.^11^–^13^abdominal hysterectomy (AH Emery et al. observed a significant (by 20%) reduction in hospitalization time for FTS compared to usual care surgery for benign laparoscopic hysterectomy.^14^ Studies by Meyer et al. and Yoong et al. also showed that FTS was associated with a decreased LOS in cancer patients who underwent gynecologic laparotomy and vaginal hysterectomy, respectively.^15^,^16^ This observed difference in LOS may be explained by the primary objective of FTS as a multimodal approach to patient care: to prevent or minimize factors that can negatively affect patient recovery throughout the entire perioperative period, such as discomfort levels, stress and anxiety, inadequate pain management, nutrition etc. Traditional preoperative preparation requires extended preoperative fasting to minimize the risk of aspiration during the procedure. However, recent studies have indicated that prolonged fasting and dehydration increase the discomfort of the patients, elevate their surgical anxiety, raise the likelihood of postoperative hypoglycemia and insulin resistance, disrupt electrolyte balance, and intensify the postoperative stress response.^5^ Numerous studies have demonstrated a clear correlation between preoperative anxiety and tension and postoperative pain, which directly influences the recovery process for patients, and showed that preoperative psychological counselling is associated with a significant reduction in anxiety levels and better coping mechanisms of the patients 6,17,18.

Our study indicated a significant decrease in VAS pain scores on the day of the surgery in the FTS group compared to the usual care group, with no difference in reported pain 24 hours after the operation. Intraoperative FTS nursing protocol employs a multimodal analgesic approach for effective pain management. This approach combines various analgesic techniques and medications, including regional anesthesia methods like epidural or spinal anesthesia, non-opioid analgesics, local anesthetics, and opioids administered through different routes (oral, intravenous, or intramuscular). The aim is to achieve optimal pain control while minimizing opioid-related side effects.^7^ Short-acting anesthetics during the surgery are utilized to promote rapid emergence from anesthesia and to reduce overall recovery room time.^8^ Our observation that FTS is associated with markedly lower VAS scores can at least partially explain shorter hospital stays after FTS. Since effective postoperative pain management is a vital part of the FTS plan, precise analgesic measures during FTS can significantly reduce postoperative pain perception of the patients, improve their overall discomfort, and encourage earlier mobilization and walking, thereby reducing the risk of postoperative cardiopulmonary complications and shortening hospital stays.^19^ Other studies have also demonstrated the pain-reducing effects of the FTS, and emphasized that a well-established multimodal approach in general is a key to achieving a shorter hospital stay.^10^,^20^ In addition to pain reduction, FT protocol effectively mitigates such elements as pre-operative hunger, fear and anxiety, diminishing stress reactions and lowering the likelihood of complications.^21^,^22^

Another important component of FTS is maintaining a normal body temperature, which is fundamental for metabolic processes and normal bodily functions. Surgery and anesthesia often lead to decreased body temperature due to surgical body exposure and anesthesia-led suppression of the hypothalamic thermoregulation center. Mild to moderate intraoperative hypothermia significantly raises the risk of wound infections and increases the overall oxygen demand during postoperative recovery.^23^,^24^ Intraoperative fluid therapy in FTS aims to maintain body fluid homeostasis and prevent organ dysfunction resulting from either excessive fluid overload or inadequate perfusion.^25^

We observed acceleration of postoperative bowel recovery and shorter mobilization time in the FTS group of patients. Postoperative FTS nursing focuses on early enteral feeding and early mobilization as key component of rapid rehabilitation surgery. Studies have demonstrated that early mobilization improves visceral nerve regulation, facilitates intestinal function recovery, reduces exhaustion time, alleviates or eliminates concerns related to postoperative abdominal distension, and enhances patient comfort.^9^ Our results support previous observations of faster postoperative bowel function recovery and faster patient mobilizations related to the modes of anesthesia and analgesia that are used in patients who undergo FTS.^26^-^28^

Current review by Scheib et al. indicated that for various types of gynecologic surgeries both FTS and standard care protocol were associated with similar rates of complications.^29^,^30^ In our study, FTS was associated with significantly lower incidence of postoperative complications compared to usual care. However, this difference became insignificant when complications were analyzed individually. We may speculate that the discrepancy between our results and the previous research may be explained by the combined accumulated effect of numerous complications.

### Limitations:

It is a single-center study with retrospective case-control design. Additionally, we analyzed data of a relatively small number of patients with benign gynecological conditions. This may impact the robustness and the generalizability of our results. Further prospective studies with larger patient samples and various gynecological conditions are needed.

## CONCLUSION

Our results indicate that implementing FTS protocol in laparoscopic gynecological surgeries for benign conditions reduces LOS without increasing postoperative complications or compromising patient satisfaction. Therefore, FTS protocol may be safely implemented as a routine practice in laparoscopic gynecological surgeries for benign conditions.

### Authors’ contributions:

**HZ:** Conceived and designed the study.

**HZ** and **XX:** Collected the data and performed the analysis.

**HZ:** Was involved in the writing of the manuscript and is responsible for the integrity of the study.

All authors have read and approved the final manuscript.
